# Erythraeid mites (Prostigmata, Erythraeidae) from Saudi Arabia, description of three new species and a new record

**DOI:** 10.3897/zookeys.445.7861

**Published:** 2014-10-13

**Authors:** Muhammad Kamran, Fahad J. Alatawi

**Affiliations:** 1 Acarology Laboratory, Department of Plant Protection, College of Food & Agriculture Sciences, King Saud University, Riyadh 11451, P.O. Box 2460, Saudi Arabia

**Keywords:** *Balaustium*, *Charletonia*, *Erythraeus*, Riyadh

## Abstract

Three erythraeid genera *Balaustium* von Heyden, *Charletonia* Oudemans, and *Erythraeus* Latreille (Trombidiformes: Prostigmata) are reported for first time from Saudi Arabia based on three new larval species, *Balaustium yousifi* **sp. n.**, *Charletonia bahaensis* **sp. n.**, and Erythraeus (Erythraeus) uhadi **sp. n.** and one new record Erythraeus (Zaracarus) lancifer Southcott. All the three new species are described and illustrated from larvae.

## Introduction

Mites of the family Erythraeidae (Trombidiformes: Prostigmata) are generally predators at postlarval stages, feeding upon various arthropods. However larvae of most erythraeids are parasites of different arthropods including insects e.g. bugs, grasshoppers, flies, aphids, etc. (Southcott 1961, 1991; Goldarazena et al. 2000; Gerson et al. 2003; Saboori and Cobanoglu 2010).

The genus *Erythraeus* Latreille comprises two subgenera, *Erythraeus* Latreille, 1806 and *Zaracarus* Southcott, 1995. The subgenus *Erythraeus* includes 93 species. Among these, 45 species are known from larvae (Khanjani et al. 2012; Mąkol and Wohltmann 2012, 2013). The subgenus *Zaracarus* includes 27 species that all have been described from larvae (Mąkol and Wohltmann 2012, 2013). More than 50% of all larval species of subgenus *Erythraeus* have been recorded as parasites on Heteroptera, Thysanoptera, Neuroptera and other insects whereas others were captured free living on herbaceous plants (Haitlinger 2012; Khanjani et al. 2010, 2012; Kamran et al. 2013; Stroiński et al. 2013).

The genus *Charletonia* Oudemans comprises 117 species: two species described from both larvae and post larval stages; 92 species described only from larvae, and 23 species known only from post larval stages (Haitlinger 2007, Beron 2008; Mąkol and Wohltmann 2012, 2013). Most larval species of this genus were recorded as parasites on Orthoptera and Heteroptera (Haitlinger 2004a; Mayoral and Barranco 2011; Saboori et al. 2012; Haitlinger et al. 2014), however some larval species were recorded free living on herbaceous plants (Haitlinger 2004a, b; Hakimitabar and Saboori 2011). The free living larvae might be collected at early larval period while searching different hosts on herbaceous plants.

The genus *Balaustium* von Heyden widespread in the world, comprises 36 nominal species: 5 species described from both larval and post- larval stages, 17 described only from post larval stages, and 14 species based only on larvae (Mąkol et al. 2012; Mąkol and Wohltmann 2012). Larvae of *Balaustium* were generally collected from plants (Mayoral and Barranco 2009; Mąkol et al. 2012). Only *Balaustium wratislaviensis* Haitlinger, 1996 was collected from different vertebrates species (Passeriformes: Paridae) (Haitlinger 1996). Family Erythraeidae is very poorly known in Saudi Arabia. Previously only *Leptus tammuzi* Haitlinger, 1994 was reported from this country (Haitlinger 1994). In this study, three genera, *Balaustium*, *Charletonia* and *Erythraeus* are reported for the first time from Saudi Arabia with three new species viz. *Balaustium yousifi* sp. n., *Charletonia bahaensis* sp. n. and Erythraeus (Erythraeus) uhadi sp. n. and one new record Erythraeus (Zaracarus) lancifer Southcott.

## Materials and methods

Three regions of Saudi Arabia, Al-Riyadh, Al-Madina and Baha, were surveyed for the collection of erythraeid mites during the years 2012–2013. Two collection methods were used: i) different plant parts were shaken over pieces of white paper and the mites were transferred using camel hair brush into 70% alcohol; ii) Tullgren funnels were used to extract mites from plant material brought to the laboratory. Mites parasitic on different insects were collected and preserved along with their hosts. Later, the mites were detached from their hosts under the stereomicroscope (Olympus®, SZX10, Japan). The collected mite specimens were cleared in Nesbitt’s fluid for 10–12 h. Subsequently, the specimens were mounted on slides in Hoyer’s medium, and dried in oven at 40 °C for one week. The mounted specimens were examined under a phase-contrast microscope (DM2500, Leica®, Germany). Temfig illustrations were either drawn with pencil by using a drawing tube (Olympus®, Japan) attached to the microscope, or different body parts of mites were pictured with an Auto-montage Software System (SYNCROSCOPY®, Cambridge, UK) attached to the microscope. Final processing of drawings was done in Adobe Illustrator (Adobe Systems Incorporated, USA). The terminology used in this study follows that of Haitlinger and Saboori (1996). All measurements are given in micrometers. The measurements in description refer to the holotype followed by as a range of paratypes in parenthesis.

## Results and discussion

### Family Erythraeidae Robineau-Desvoidy

#### Subfamily Erythraeinae Robineau-Desvoidy

##### Genus Erythraeus

Taxon classificationAnimaliaProstigmataErythraeidae

Latreille

###### Type species.

*Acarus phalangoides* (de Geer), by original designation.

##### Erythraeus (Erythraeus) uhadi sp. n.

Taxon classificationAnimaliaProstigmataErythraeidae

http://zoobank.org/D69C9E7F-7869-4556-9ABE-8485E7F66DEF

Figs 1 –13

###### Diagnosis

(n=6). fn Bfe 3-3-3, IP 2519–2597, fnTi 14-15-15, fD 32, fV 10, AL 90-97, AP 32–35, PSE 80–87, Ti III 279-289, Ti II 180-196, Genu III 143-149.

###### Description.

**Figures 1–4.**
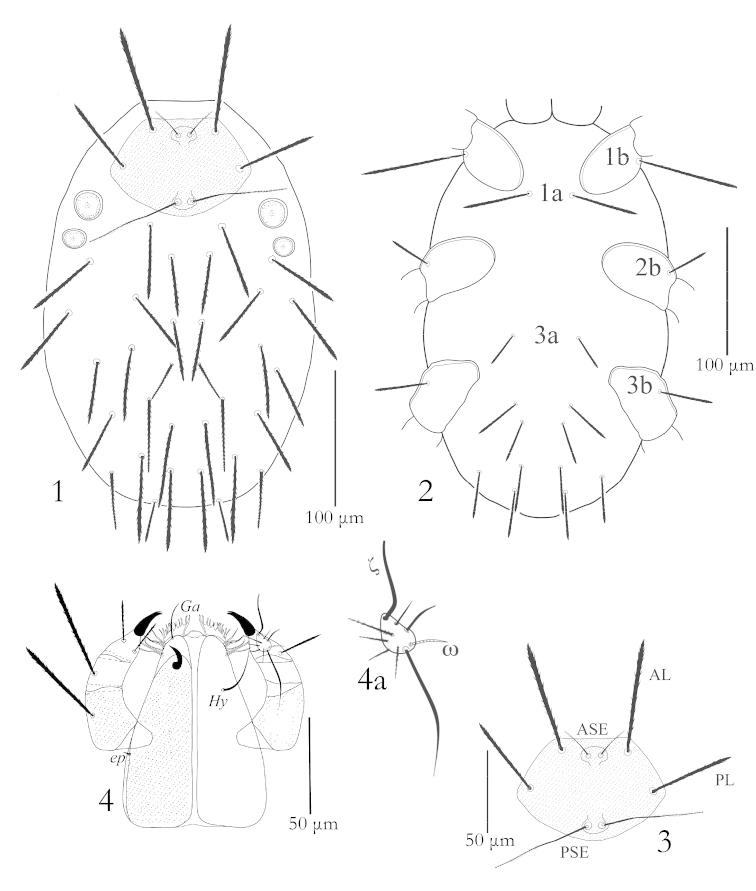
Erythraeus (Erythraeus) uhadi sp. n., (Larva): **1** Dorsum **2** Venter **3** Scutum **4** Gnathosoma (left dorsal view, right ventral view) **4a** Palptarsus.

(Holotype larva):

Dorsum: Prodorsal scutum with two pairs of sensilla (ASE and PSE) and two pairs of setae (AL and PL). AL located slightly anterior to ASE bases, PSE present at posterior pole of scutum, Posterior pair of sensilla (PSE) more than three times longer than anterior pair ASE, both finely ciliated on their distal halves. Cuticular lines surround both sensilla. AL longer than PL, both with long dense barbs on their entire lengths. Prodorsal scutum almost pentagonal in shape, straight anteriorly, round posteriorly, widest at the level of PL setae (Fig. 3). Two pairs of eyes present at the level of posterior end of scutum dorsolaterally on idiosoma, anterior pair 24 (22–24) across, posterior pair 14 (13–14) across. Dorsal setae on idiosoma, 16 pairs (fD = 32), barbed and ranging in lengths from 29–61 (28–64)(Fig. 1).

Venter: Idiosoma ventrally bears setae *1a* between coxae I, setae *3a* slightly anterior to the area between coxae III; *1a* 50 (48–54), 3a 28 (28–32) long; opisthogaster behind the coxae III with 10 setae (fV=10). All ventral setae with dense barbs. NDV = 32+10 = 42 (Fig. 1B). Coxae I-III each with one coxalae; all coxalae barbed. Coxalae *1b* three times longer than *2b* (Fig. 2).

Gnathosoma: Infracapitulum with one pair of nude hypostomal setae (*Hy*) 30 (30–34) and nude galealae (*Ga*) 23 (21–24), supracoxalae present, very small, peg-like. Palp five segmented, palpfemur and genu each with one barbed seta, palptibia with three barbed setae, tibial claw bifurcate. Palptarsus with one eupathidium, one solenidion, two smooth and four barbed setae including one long seta (Figs 4, 4a). Palp setal formula: fPp: 0-B-B-BBB_2_-NNBBBBζω.

**Figures 5–7.**
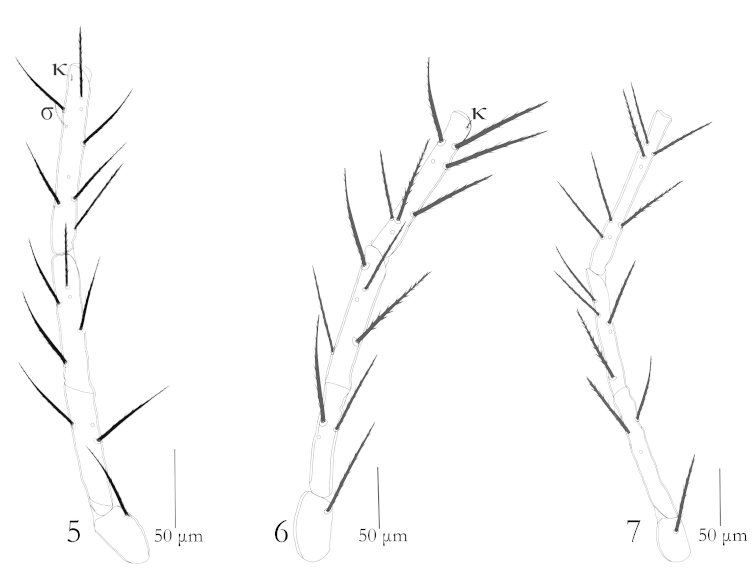
Erythraeus (Erythraeus) uhadi sp. n., (Larva): **5** Trochanter, femur & genu I **6** Trochanter, femur & genu II **7** Trochanter, femur & genu III.

**Figures 8–13.**
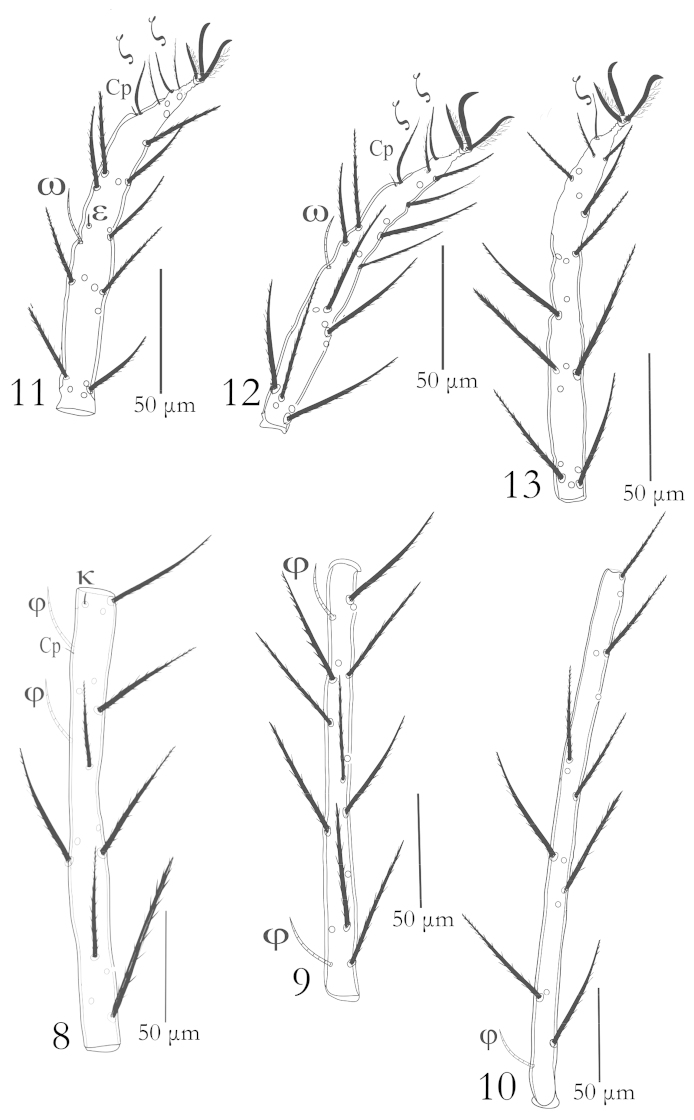
Erythraeus (Erythraeus) uhadi sp. n., (Larva): **8** Tibia I **9** Tibia II **10** Tibia III **11** Tarsus I **12** Tarsus II **13** Tarsus III.

Legs: Legs seven segmented with divided femora, all legs longer than body length; leg III the longest one, Tarsi terminate into two lateral claws and a claw like empodium. Chaetotaxy of leg segments: coxae 1–1–1; trochanters 1–1–1; basifemora 3–3–3; telofemora 5–5–5; genua 8+1σ+1κ – 8+1κ – 8; tibiae 14 + 2φ+ 1*Cp* + 1κ – 15 + 2φ – 15+1φ; tarsi 22 + 1ω + 1ε + 1*Cp* + 2ζ – 20+ 1ω + 1*Cp* + 2ζ – 20 + 1ζ (Figs 5–13).

###### Etymology.

The specific epithet is derived from the name of famous mountain "Uhad", where holotype larva was collected.

###### Type material.

Holotype larva was collected from the mountain “Uhad”, Al-Madina, Saudi Arabia, 24°30.086'N, 39°36.41'E, on 23 February, 2013, coll. M. Kamran), parasitizing tamarix leafhopper, *Opseius* sp. (Hemiptera: Cicadellidae), from *Tamarix* sp. (Tamaricaceae). Paratypes 4 larvae, collection data same as holotype, while one paratype was collected from Wadi-e-Hanifa near Arqa over bridge, Riyadh, Saudi Arabia, 24°41.354'N, 46°37.042'E, on 14 April, 2013, from *Tamarix* sp. in association with the same host, coll. M. Kamran. Holotype and 4 paratypes (P2, P3, P4, P5) are deposited in the King Saud University Museum of Arthropods (KSMA) and Acarology Laboratory, Department of Plant Protection, College of Food and Agriculture Sciences, King Saud University. One paratype (P1-accession no. Acy: 14/47) has been deposited at the Agriculture Research Council, Plant Protection Research Institute, Biosystematics Division, Pretoria (ARC-PPRI), South Africa.

###### Remarks.

Erythraeus (Erythraeus) uhadi sp. n. belongs to a group of species of subgenus *Erythraeus* that share the following combination of characters: basifemoral setal formula 3–3–3, tibia I with 14 normal setae, Ti III 270–334, Ti II 170–210, genu III 120–200. This group includes 7 species: Erythraeus (Erythraeus) flavopictus Kawashima, 1961; Erythraeus (Erythraeus) sabrinae Haitlinger & Saboori, 1996; Erythraeus (Erythraeus) southcotti Goldarazena & Zhang, 1998; Erythraeus (Erythraeus) ankaraicus Saboori et al., 2004; Erythraeus (Erythraeus) zhangi Haitlinger, 2006; Erythraeus (Erythraeus) hilarae Haitlinger, 2010, Erythraeus (Erythraeus) chrysoperlae Khanjani et al., 2012 (Kawashima 1961; Haitlinger and Saboori 1996; Goldarazena and Zhang 1998; Saboori et al. 2004; Haitlinger 2006a, 2010; Khanjani et al. 2012). Erythraeus (Erythraeus) uhadi sp. n. differs from Erythraeus (Erythraeus) flavopictus by shorter ASE (22–25 vs. 55), shorter W (99–108 vs. 153), shorter IP (2519–2597 vs. 2944), shorter AP (32–35 vs. 59), fD (32 vs. 42); from Erythraeus (Erythraeus) sabrinae by shorter AP (32-35 vs. 52), fD (32 vs. 62), fV (10 vs. 28), shorter W (99–105 vs. 132), shorter AW (44–47 vs. 60), shorter PW (81–85 vs. 110); from Erythraeus (Erythraeus) southcotti by shorter AP (32–35 vs. 48–50), longer PaScGed (50-54 vs. 25-30); fD (32 vs. 46), fV (10 vs. 16), fnTa (21–20–20 vs. 26–23–24); from Erythraeus (Erythraeus) zhangi by shorter L (69–81 vs. 96–128), shorter W (99–108 vs. 126–148); shorter GL (106–111 vs. 140–166), shorter IP (2519–2597 vs. 2622–3198), fD (32 vs. 86), fV (10 vs. 20); Erythraeus (Erythraeus) ankaraicus by fnTa (21–20–20 vs 25–22–24), fD (32 vs. 41), fV (10 vs. 18), AL (90–97 vs. 65–78), AP (32–35 vs. 41-48); from Erythraeus (Erythraeus) hilarae by shorter L (69–81 vs. 110), shorter W (99–108 vs. 128), shorter ISD (49-53 vs. 68), shorter GL (106–111 vs. 130), fV (10 vs. 16), fnTi (14-15-15 vs. 14–14–14) and from Erythraeus (Erythraeus) chrysoperlae by fV (10 vs. 14), fnTa (21–20–20 vs. 27–23–24), longer AL (90–97 vs. 70), shorter AP (32–35 vs. 50), shorter GL (106-111 vs. 150).

**Table 1.** Metric data of Erythraeus (Erythraeus) uhadi sp. n. larva (holotype and 5 paratypes).

Ch.	H	P1	P2	P3	P4	P5	Ch.	H	P-1	P2	P3	P4	P5
IL	302	300	305	307	298	297	Ta I(H)	16	15	16	15	16	16
IW	195	197	195	200	194	199	Ti I	205	206	205	210	211	207
L	71	73	70	74	69	81	Ge I	185	183	185	190	193	186
W	105	103	102	108	106	99	Tfe I	113	111	115	112	116	110
AW	44	45	44	48	46	47	Bfe I	105	106	103	107	110	104
PW	81	83	82	85	81	85	Tr I	44	45	46	43	47	44
AA	11	11	11	12	11	12	Cx I	35	34	36	34	36	35
SB	13	13	13	14	13	14	Leg I	829	828	834	843	853	826
ISD	50	52	49	53	53	51	Ta II(L)	136	138	135	139	141	134
AP	34	33	35	35	32	35	Ta II(H)	15	15	15	14	15	15
AL	92	90	93	97	91	95	Ti II	189	187	189	180	196	192
PL	63	61	62	60	65	60	Ge II	126	127	129	124	131	122
ASE	23	24	25	22	23	22	Tfe II	110	108	113	107	113	110
PSE	81	80	82	87	81	84	Bfe II	95	97	96	98	94	94
DS	29–61	29–62	28–61	30–64	30–63	29–62	Tr II	50	52	50	48	54	53
PDS	29–61	29–62	29–61	29–64	29–63	29–62	Cx II	63	65	63	60	61	61
*1a*	50	52	53	54	48	50	Leg II	769	774	775	756	790	766
*3a*	28	29	28	32	30	31	Ta III(L)	154	152	156	150	157	153
*1b*	100	99	102	105	100	103	Ta III(H)	15	15	15	14	15	15
*2b*	33	32	30	35	32	34	Ti III	286	287	279	287	289	283
*3b*	38	37	36	40	39	38	Ge III	148	149	146	146	143	144
*Hy*	30	31	30	34	32	30	Tfe	113	114	110	112	116	113
*Ga*	23	22	21	24	23	22	Bfe	123	123	125	126	128	122
G L	107	110	108	111	106	107	Tr III	50	53	52	50	53	51
PaScFed	50	52	51	54	51	49	Cx III	66	67	65	66	68	67
PaScGed	52	54	52	56	50	53	LegIII	940	945	933	937	962	933
Ta I(L)	142	143	144	147	140	140	IP	2538	2547	2542	2519	2597	2525

Ch = Character, H = Holotype, P = Paratype

##### Subgenus *Zaracarus* Southcott

###### Erythraeus (Zaracarus) lancifer

Taxon classificationAnimaliaProstigmataErythraeidae

Southcott

Erythraeus (Zaracarus) lancifer Southcott, 1995: 223.

####### Material examined.

Six larvae, Baha, Saudi Arabia, 20°7.918'N, 41°24'69'E on 24 April, 2013, coll. M. Kamran, parasitizing tamarix leafhopper, *Opseius* sp. (Hemiptera: Cicadellidae); two larvae were collected as free living on *Setaria viridis* L. (Poaceae) from the same locality and date.

####### Remarks.

The type specimens were collected from a fly (Diptera, Dolichopodidae) Nr Pina, Zaragoza Province, Spain (Southcott 1995). This species has been hitherto only recorded from Spain. Present samples constitute a new record for Asia.

**Table 2.** Metric data of Erythraeus (Zaracarus) lancifer larva (measurements of 4 specimens in range).

Ch.		Ch.		Ch.		Ch.	
IL	344–355	PSE	73–79	Ti I	228–234	Tr II	62–66
IW	230–238	DS	55–72	Ge I	164–167	Cx II	67–72
L	91–97	*1a*	41–44	Tfe I	110–115	Leg II	851–891
W	145–151	*3a*	30–34	Bfe I	112–116	Ta III(L)	156–163
AW	41–45	*1b*	88–94	Tr I	54–56	Ta III(H)	16
PW	110–115	*2b*	29–32	Cx I	63–67	Ti III	329–334
AA	20–21	*3b*	34–37	Leg I	893–923	Ge III	156–160
SB	15–15	*Hy*	30–33	Ta II(L)	137–143	Tfe	135–140
ISD	62–65	*Ga*	23–26	Ta II(H)	16–17	Bfe	129–133
AP	50–53	PaScFed	54–58	Ti II	229–236	Tr III	52–55
AL	186–197	PaScGev	67–71	Ge II	129–137	Cx III	68–72
PL	74–79	Ta I(L)	162–168	Tfe II	122–127	Leg III	1025–1057
ASE	28–30	Ta I(H)	17–18	Bfe II	105–110	IP	2769–2871

#### Subfamily Callidosomatinae Southcott

##### Genus *Charletonia* Oudemans

###### Charletonia bahaensis sp. n.

Taxon classificationAnimaliaProstigmataErythraeidae

http://zoobank.org/BEBA76A2-2E8F-4102-8BAD-714C99FE6F2A

Figs 14 –23

####### Diagnosis

(n=7). fnTi 18-18-18, fD 121–123, fV 60–61, with two hypostomalae, posterior hypostomalae barbed, galeala nude, GL 157-164, fnGe 12-12-12, four setae between coxae II & III, solenidion on genu I located distally.

####### Description of holotype larva.

(Metric data of holotype followed by as a range of six paratypes in parenthesis).

**Figures 14–17.**
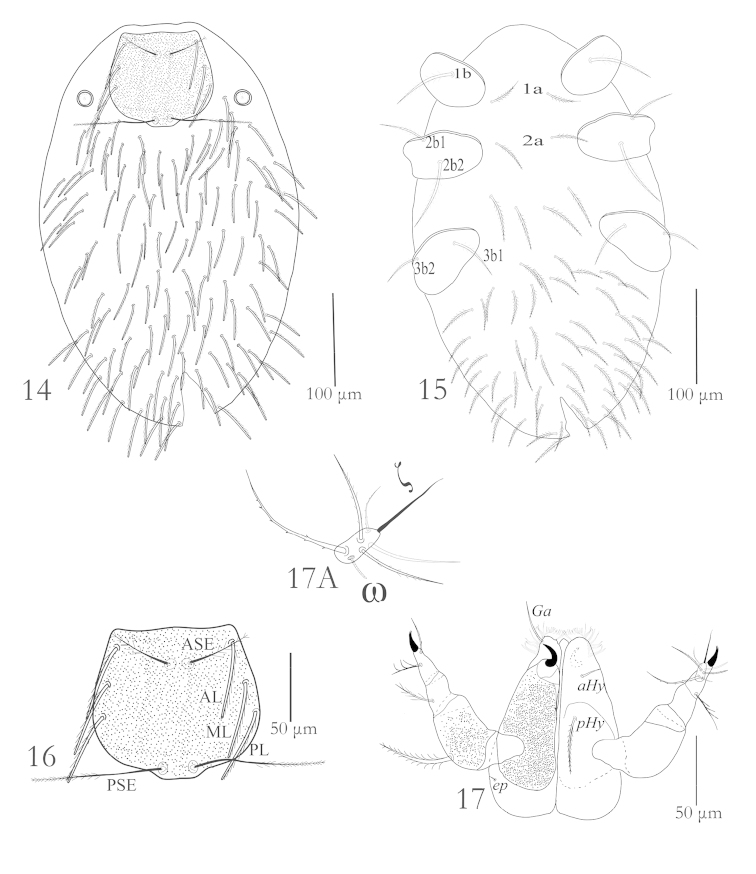
*Charletonia bahaensis* sp. n. (Larva): **14** Dorsum **15** Venter **16** Scutum **17** Gnathosoma (left dorsal view, right ventral view) **17A** Palptarsus.

Dorsum: Prodorsal scutum punctate entirely, with two pairs of sensillae (ASE, PSE) and three pairs of normal setae (AL, PL, PL). Posterior sensilla (PSE) longer than anterior ones (ASE), both finely barbed at distal halves. All three scutalae AL, ML and PL densely barbed and blunt ended, (Fig. 16). Dorsum with 123 (121–123) barbed setae (fD = 123 (121–123) with blunt tips, ranging in lengths from 45 (42–56). A pair of eyes located laterally on idiosoma posterolateral to scutum, 21 (21–23) across (Fig. 14).

Venter: Venter with intercoxal setae (*1a*) between coxae I, one pair of intercoxal setae (*2a*) between coxae II, four setae in the area between coxae II & III, 57 (56–57) setae present on opisthogaster behind the coxae III (fV = 61 (60–61). All ventral setae barbed with pointed tips except postero-marginal setae on venter which are blunt-ended (Fig. 15).

Gnathosoma: Subcapitulum with one pair of nude, spiniform galealae (*Ga*) 33 (30–34), two pairs of hypostomalae, anterior pair (*aHy*) nude, 16 (15–17), posterior pair (*pHy*) with long barbs, 45 (42–47). Chelicerae 114 (113–116), cheliceral blade 19 (18–19). Supracoxalae present, very small, peg- like. Palpfemur and genu each with one barbed seta, palptibia with three barbed setae and bifurcated claw (Fig. 17), palptarsus with one eupathidium, one solenidion, one nude and four barbed setae including long basal seta (Fig. 17A), eupathidium 25 (23–25), solenidion 7 (6–7) and long basal seta, 39 (35–40) long. Palp setal formula: 0-B-B-BBB_2_–4BNωζ.

Legs: Legs seven segmented with divided femora, all longer than body length. Tarsi I–III terminate in two lateral claws and claw like empodium.

**Figures 18–20.**
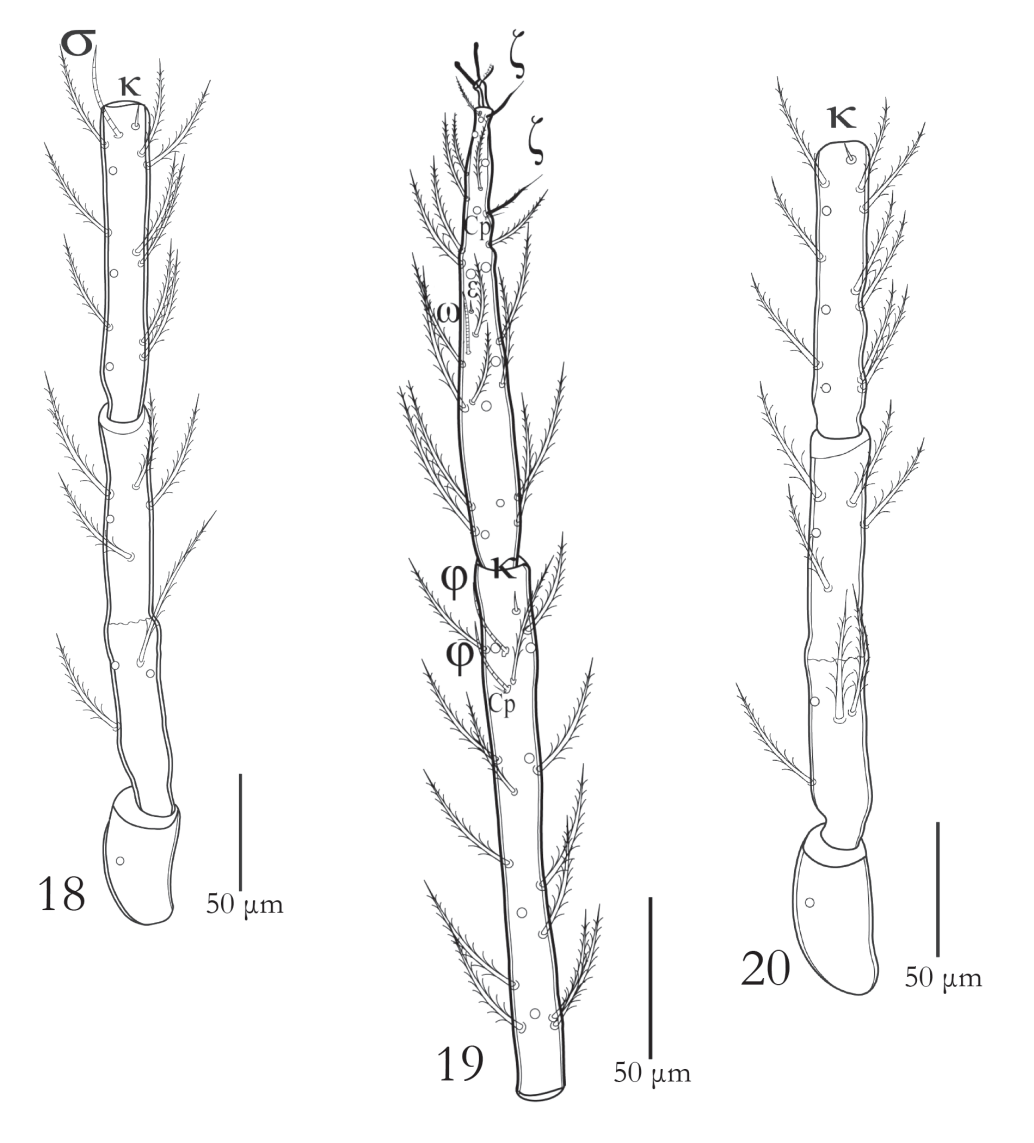
*Charletonia bahaensis* sp. n. (Larva): **18** Trochanter, femur & genu I **19** Tibia & Tarsus I **20** Trochanter, femur & genu II.

**Figures 21–23.**
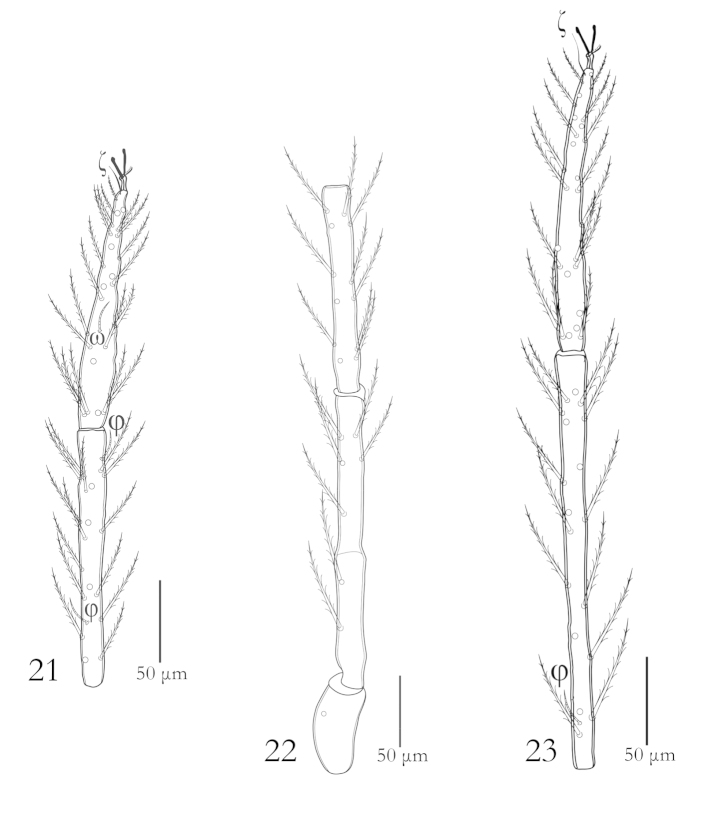
*Charletonia bahaensis* sp. n. (Larva): **21** Tibia & Tarsus II **22** Trochanter, femur & genu III **23** Tibia & Tarsus III.

Leg setal formula: Cx: 1-2-2; Tr: 1-1-1; Bfe: 4-4-2; Tfe: 5-5-5; Ge: 12+1σ+1κ – 12+ 1κ – 12; Ti: 18+2φ + 1Cp+ 1κ – 18+ 2φ –18 + 1φ; Ta: 27+ 1ω + 1ε + 1Cp + 2ζ – 26 + 1ω + 1ζ – 27 + 1ζ (Figs 18–23).

####### Etymology.

The specific epithet is derived from the city name “Baha” (in Saudi Arabia) where it was collected.

####### Type material.

Holotype and 6 paratype larvae, from blue alfalfa aphid, *Acyrthosiphon kondoi* Shinji (Hemiptera: Aphididae), infesting alfalfa plants, *Medicago sativa* L., Baha, Saudi Arabia, 19°59.807'N, 41°25.715'E, on 25 April, 2013, coll. M. Kamran. Holotype and 5 paratypes (P2, P3, P4, P5, P6) are deposited in the King Saud University Museum of Arthropods (KSMA) and Acarology Laboratory, Department of Plant Protection, College of Food and Agriculture Sciences, King Saud University. One paratype (P1- accession no. Acy: 14/46) has been deposited at the Agriculture Research Council, Plant Protection Research Institute, Biosystematics Division, Pretoria (ARC-PPRI), South Africa.

####### Remarks.

*Charletonia bahaensis* sp. n. belongs to the species group of genus *Charletonia* with four setae between coxae II & III, solenidion placed distally on genu I, fn Ge 12–12–12, Ti III 200–260 and two hypostomalae. This group includes 11 species: *Charletonia areolata* (Trägårdh, 1908); *Charletonia froggatti* Oudemans, 1910; *Charletonia feideri* Southcott, 1966; *Charletonia rageaui* Southcott, 1966; *Charletonia paolii* Southcott, 1966; *Charletonia banksi* Southcott, 1966; *Charletonia enghoffi* Southcott, 1991; *Charletonia hunanensis* Zheng, 1996; *Charletonia lombokensis* Haitlinger, 2006; *Charletonia grandpopensis* Haitlinger, 2007 and *Charletonia salazari* Mayoral & Barranco, 2011 (Southcott 1966, Southcott 1991, Zheng 1996, Haitlinger 2006b, 2007, Mayoral and Barranco 2011). The new species differs from *Charletonia areolata* by fD (121–123 vs. 97), fV (60–61 vs. 42), setae on Ti III (18 vs. 19), Ti III (231–242 vs. 259), Ti I (175–183 vs. 199), Ge I (127–135 vs. 157), Galealae (nude vs. ciliated); from *Charletonia froggatti* by fD (123 vs. 64), fV (60–61 vs. 37), fnTi (18–18–18 vs. 14–14–18); from *Charletonia feideri* by fD (121–123 vs. 86), fV (61 vs. 44), setae on Ti III (18 vs. 19), Ti I (173–184 vs. 138–159), Ge III (140–148 vs. 121), Ge I (127–135 vs. 112–125), Ta I (158–166 vs. 129–140); from *Charletonia rageaui* by fD (121–123 vs. 94), fV (61 vs. 54), fnTi (18–18–18 vs. 18–18–19), Ta I (158–166 vs. 142–149); from *Charletonia paolii* by fD (121–123 vs. 98), setae on Ti III 18 vs. 19), posterior hypostomalae (barbed vs. nude), W (114–118 vs. 98), PL (49–55 vs. 36–43), Ta I (158–166 vs. 137), galealae (nude vs. barbed), Ta III (165-177 vs. 133); from *Charletonia banksi* by fD (121–123 vs. 97), fV (60–61 vs. 46), setae on Ti III (18 vs. 19), Ge III (140–148 vs. 125), galealae (nude vs. barbed), leg I (741–781 vs. 725), leg II (694–716 vs. 660), leg III (869–911 vs. 790); from *Charletonia enghoffi* by fD (121–123 vs. 52), fV (60–61 vs. 40), setae on Ti I (18 vs. 17), posterior hypostomalae (barbed vs. nude), PSE (87–95 vs. 116–129), ASE (48–51 vs. 70–75); *Charletonia hunanensis* by fD (121–123 vs. 73), fV (60–61 vs. 47), setae on Ti II (18 vs. 21), Ge III (140–148 vs. 125), setae on Tfe (5 vs. 6); from *Charletonia lombokensis* by fD (121–123 vs. 74), fV (60–61 vs. 40), setae on Ti II (18 vs. 17), fnBfe (4–4–2 vs. 3–3–2), PW (106–113 vs. 50), ASE (48-54 vs. 22), PSE (87-95 vs. 36); from *Charletonia grandpopensis* by fD (121–123 vs. 60), fV (60–61 vs. 43), setae on Ti II (18 vs. 17), setae on Ti III (18 vs. 17), ASE (ciliated vs. nude), DS (42–56 vs. 68–72), Ta I (158–166 vs. 130–134), GL (155–164 vs. 96–108), galealae (nude vs. barbed); from *Charletonia salazari* by fD (121–123 vs. 76), fV (60–61 vs. 28), fnTi (18–18–18 vs. 15–16–16), ISD (71–78 vs. 54–63), AL (50–56 vs. 67–72), AP (48–52 vs. 68–72). In brief the new species can be differentiated from all other species of this group by having fD 123, fV 61 and fn Ti 18–18–18. All other species of this group have dorsal setae less than 100.

**Table 3.** Metric data of *Charletonia bahaensis* sp. n. larva, holotype and 6 paratypes (in range).

Ch.	H	P1	P2	P3	P4	P5	P6	Ch.	H	P1	P2	P3	P4	P5	P6
IL	441	436	439	435	430	442	441	PaScFed	58	55	57	58	57	55	59
IW	280	285	275	272	276	278	282	PaScGev	32	30	30	29	33	29	33
L	110	112	109	108	110	106	113	Ta I(L)	164	160	158	166	165	159	165
W	116	117	118	114	116	115	117	Ta I(H)	16	15	17	16	16	16	17
AW	84	81	86	81	86	84	85	Ti I	181	180	178	183	175	173	184
MW	98	94	100	97	101	93	98	Ge I	132	133	127	135	130	129	135
PW	110	112	109	108	112	113	106	Tfe I	88	85	89	90	90	86	91
AA	10	10	11	10	11	10	10	Bfe I	88	86	89	90	85	84	91
SB	20	19	20	10	19	21	18	Tr I	47	49	46	47	46	46	47
ISD	75	71	78	72	77	75	71	Cx I	66	65	67	67	63	64	68
AP	49	50	52	47	50	48	49	Leg I	766	758	754	778	754	741	781
AL	54	52	51	50	54	55	56	Ta II(L)	152	146	150	154	154	150	155
ML	54	55	52	53	57	57	58	Ta II(H)	15	15	16	15	16	15	16
PL	52	51	49	50	55	53	55	Ti II	156	159	153	153	151	150	155
ASE	49	50	51	48	54	50	49	Ge II	113	111	110	114	115	110	116
PSE	93	91	90	87	95	89	95	Tfe II	78	85	77	80	75	76	81
DS	45–54	44–55	43–54	42–53	45–55	44–54	45–56	Bfe II	79	78	80	82	77	80	83
PDS	45–54	44–55	43–54	42–53	45–55	44–54	45–56	Tr II	59	60	62	58	56	57	61
*1a*	44	45	42	40	45	44	46	Cx II	74	71	73	75	70	71	74
*2a*	57	55	54	54	60	58	59	Leg II	711	710	705	716	698	694	725
*1b*	71	69	68	67	73	73	72	Ta III(L)	172	170	166	177	165	168	175
*2b1*	71	69	73	67	78	77	73	Ta III(H)	16	15	16	16	15	15	16
*2b2*	55	53	56	52	56	57	54	Ti III	237	239	233	231	242	230	241
*3b1*	55	52	57	52	57	56	53	Ge III	146	144	148	148	140	141	147
*3b* 2	46	44	47	42	48	45	42	Tfe	113	111	115	109	112	110	115
GL	161	158	163	155	164	159	157	Bfe	89	88	90	87	**90**	87	90
*pHy*	45	44	42	43	47	46	47	Tr III	59	60	56	58	59	56	60
*aHy*	16	17	16	16	17	17	15	Cx III	80	81	78	77	80	77	83
*Ga*	33	34	32	31	34	33	30	LegIII	895	893	886	887	903	869	911
								IP	2372	2361	2345	2381	2355	2304	2417

#### Subfamily Balaustiinae Grandjean

##### Genus *Balaustium* von Heyden

###### Balaustium yousifi sp. n.

Taxon classificationAnimaliaProstigmataErythraeidae

http://zoobank.org/71EF1ABE-54D9-430E-9D44-40E5E1A3B5F1

Figs 24 –29

####### Diagnosis

(n=7). Scutum present, three pairs of scutalae present off the scutum, fnTr 3–3–2, fnBfe 4–4–3, fnTi 11–11–11, PSE 66-75, IP 1294–1363, ISD 65-69, fV 60 and fD 74.

####### Description of holotype larva.

**Figures 24–26.**
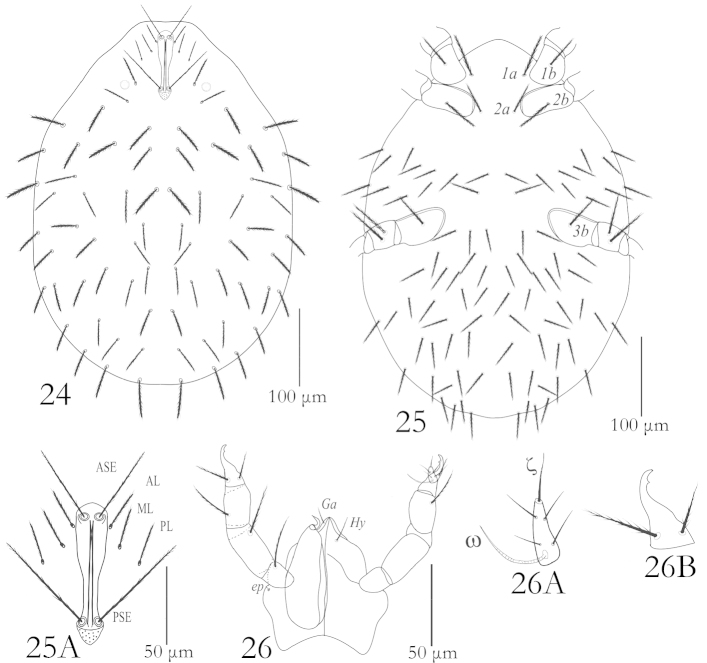
*Balaustium yousifi* sp. n. (Larva): **24** Dorsum **25** Venter **25A** dorsal scutum **26** Gnathosoma (left dorsal view, right ventral view) **26A** Palptarsus, **26B** Palptibia.

Dorsum: Idiosoma oval in shape, scutum elongate, 92 (88–95) long, 23 (21–25) wide, carries two pairs of sensilla (ASE & PSE), ASE located on anterior while PSE on posterior part of scutum, both sensilla finely barbed on their entire lengths. Crista present on scutum. Three pairs of scutalae (AL, ML, PL) present on the lateral sides of scutum, no scutalae located on scutum. AL located slightly posterior to the bases of ASE, ML lies slightly anterior to the middle of scutum and PL slightly posterior to the middle of scutum. One pair of eyes present on postero-lateral side of scutum at the level of PSE on the idiosoma, cornea of each eye 14 (13–14) in diameter. Dorsal setae on idiosoma 37 pairs, all barbed. fD = 74 (Fig. 24).

Venter: Idiosoma ventrally with one pair of sternalae *1a* between coxae I, 56 (52–57) long, one pair of setae *2a* between coxae II, 42 (41–47) long, 26 setae present in the area between coxae II & III, 60 (59–60) setae present between and behind the coxae III (fV = 86 (84–86). All ventral setae barbed (Fig. 25).

Gnathosoma: Gnathosoma with one pair of hypostomalae (*Hy*) 16 (15–17) and one pair of galealae (*Ga*) 10 (9–10), both barbed, supracoxalae present, very small, peg- like. Chelicerae 52–55 long, cheliceral blade 9 (9–10). Palp trochanter and palpfemur each with one barbed setae, palpgenu with two barbed setae (Fig. 26); palptibia withthree setae, palptarsus with four nude setae, one eupathidium and one solenidion(Fig. 26A). Palptibial claw entire with a median tooth (Fig. 26B). Eupathidium 7 (7), solenidion 16 (14–16). (Fig. 26). Palp setal formula: fPp: B-B-BB-BBN-NNNNωζ.

**Figures 27–29.**
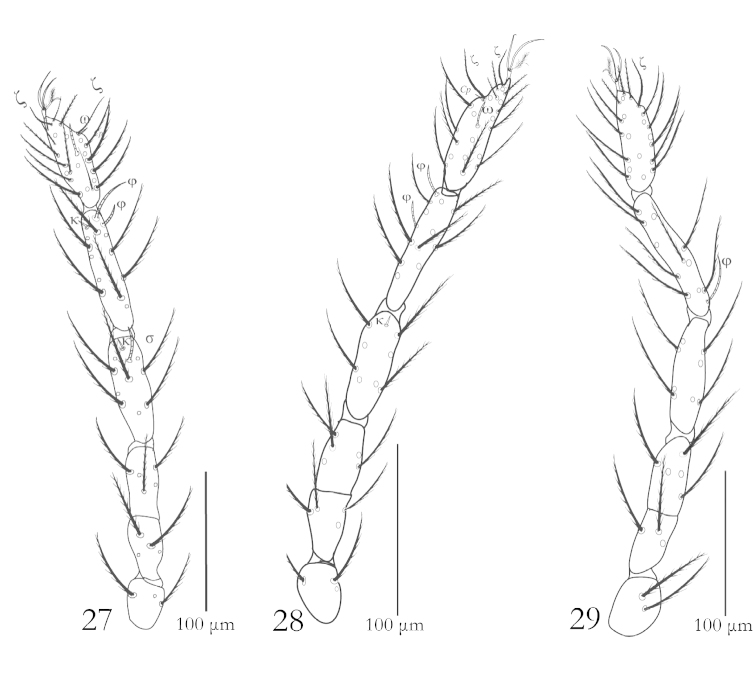
*Balaustium yousifi* sp. n. (Larva): **27** Leg I **28** Leg II **29** Leg III.

Legs: Legs seven segmented with divided femora, tarsi I–III terminated with two claws and claw-like empodium, empodium with pilose (pulvilliform) structure. Leg setal formula: leg I: Ta- ω, 2ζ, 1 Cp, 22B; Ti- 2φ,1κ, 11B; Ge- 1σ,1κ, 9B; Tfe- 5B; Bfe- 4B; Tr- 3B; Cx- 1B (Fig. 27). Leg II: Ta- ω, 1ζ, 20B; Ti- 2φ, 11B; Ge- 1κ, 8B; Tfe- 5B; Bfe- 4B; Tr- 3B; Cx- 1B (Fig. 28). Leg III: Ta- 20B; Ti- 1φ, 11B; Ge- 8B; Tfe- 5B; Bfe- 3B; Tr- 2B; Cx- 1B (Fig. 29).

####### Etymology.

The new species is named on the name of Professor Dr. Yousif Al-Duraihim.

####### Type.

Holotype larva was collected from 5 Km Taif road, Baha, Saudi Arabia, 20°7.918'N, 41°24.69'E, 24 April, 2013 (Coll. M. Kamran), from foxtail grass, *Setaria viridis* L. Paratypes six larvae, collection data same. Holotype and 6 paratypes (P1, P2, P3, P4, P5, P6) are deposited in the King Saud University Museum of Arthropods (KSMA) and Acarology Laboratory, Department of Plant Protection, College of Food and Agriculture Sciences, King Saud University. One paratype (P1- accession no. Acy: 14/45) has been deposited at the Agriculture Research Council, Plant Protection Research Institute, Biosystematics Division, Pretoria (ARC-PPRI), South Africa.

####### Remarks.

*Balaustium yousifi* sp. n. closely resembles with *Balaustium florale* Grandjean. However it differes from *Balaustium florale*. by length of PSE (66–75 vs. 40–48); IP (1294–1363 vs. 850–988); ISD (64–69 vs. 42–48); fD (74 vs. 82). The new species can be distinguished from *Balaustium bisculatae* Mayoral & Barranco by shorter ISD (65–69 vs. 56), fD (74 vs. 95), longer AL (28–32 vs. 24), longer TiIII (89–97 vs. 72–75), longer IP 1294–1348 vs. 1014–1042.

**Table 4.** Metric data of *Balaustium yousifi* sp. n. larva (holotype and 6 paratypes).

Ch.	H	P-1	P2	P3	P4	P5	P6	Ch.	H	P1	P2	P3	P4	P5	P6
IL	471	478	466	475	459	465	460	Ta I(H)	23	22	22	22	23	24	22
IW	345	336	355	349	332	340	342	Ti I	89	92	88	86	94	93	86
L	92	95	89	88	89	95	91	Ge I	92	88	89	90	93	93	86
W	23	22	24	23	24	25	21	Tfe I	54	50	55	53	56	49	54
AW	28	28	29	30	27	30	28	Bfe I	59	60	61	58	62	58	55
MW	39	37	40	39	36	41	41	Tr I	32	31	33	30	34	34	30
PW	64	66	61	62	60	63	65	Cx I	65	62	66	64	60	61	60
SBa	12	12	11	12	12	12	12	Leg I	479	473	479	463	484	470	462
SBp	16	15	16	15	16	15	16	Ta II(L)	79	82	76	75	83	79	81
ISD	68	66	69	65	64	66	68	Ta II(H)	22	23	22	22	23	21	21
AL	30	28	30	32	29	32	31	Ti II	77	75	79	76	76	77	76
ML	30	30	29	30	28	29	32	Ge II	71	72	73	68	74	69	68
PL	34	35	36	34	33	34	32	Tfe II	44	41	39	40	42	45	46
ASE	53	50	55	52	50	56	51	Bfe II	38	37	34	35	37	39	40
PSE	72	69	74	66	70	75	71	Tr II	36	38	39	39	42	43	35
DS	28–42	27–43	29–43	28–40	26–40	28–44	30–42	Cx II	60	58	60	60	65	63	64
PDS	33–42	34–43	33–43	31–40	30–40	29–44	34–42	Leg II	405	403	400	393	419	415	410
1a	56	54	52	56	52	57	57	Ta III(L)	82	81	79	79	83	85	78
*1b*	45	42	45	46	41	47	41	Ta III(H)	19	19	20	19	20	19	20
*2b*	49	44	50	48	44	46	46	Ti III	94	96	92	89	97	92	91
*3b*	47	47	46	47	45	45	48	Ge III	78	75	79	74	77	79	77
GL	88	90	88	85	85	92	82	Tfe III	51	51	55	55	54	56	50
PaScFed	33	34	35	33	31	35	31	Bfe III	51	49	54	49	55	56	54
PaScFev	22	21	20	23	20	23	22	Tr III	35	34	36	33	37	36	33
PaScGed	24	25	23	24	22	26	22	Cx III	61	64	58	59	57	59	60
PaScGev	18	17	18	19	17	20	18	Leg III	452	450	453	438	460	463	443
Ta I(L)	88	90	87	82	85	91	91	IP	1336	1326	1332	1294	1363	1348	1315

## Supplementary Material

XML Treatment for Erythraeus

XML Treatment for Erythraeus (Erythraeus) uhadi

XML Treatment for Erythraeus (Zaracarus) lancifer

XML Treatment for Charletonia bahaensis

XML Treatment for Balaustium yousifi
